# Complement expression profiles in human glomerular mesangial cells, endothelial cells, podocytes and proximal tubular epithelial cells

**DOI:** 10.3389/fimmu.2026.1881549

**Published:** 2026-09-01

**Authors:** Yueming Gao, Qi Li, Yue Wang, Zhenling Deng

**Affiliations:** Department of Nephrology, Peking University Third Hospital, Beijing, China

**Keywords:** complement, endothelial cells, mesangial cells, podocytes, proximal tubular epithelial cells

## Abstract

**Background:**

Local expression of complement components in the kidney has been reported sporadically in both diseased and normal kidneys. This study aimed to comprehensively characterize the expression of complement components in human glomerular mesangial cells (GMCs), glomerular endothelial cells (GECs), podocytes, and proximal tubular epithelial cells (PTECs) in non-diseased renal tissue.

**Methods:**

Complement expression in cultured human renal intrinsic cells was initially evaluated using reverse transcription polymerase chain reaction and immunofluorescence staining. These findings were further examined using publicly available single-cell RNA-sequencing datasets and 10×Genomics single-cell RNA sequencing of non-diseased human kidney tissue. The analyses focused on complement components involved in the initiation of the classical, lectin, and alternative pathways, as well as components shared among these activation pathways, terminal pathway components, complement regulators, and complement receptors.

**Results:**

Complement components unique to the initial phase for classical pathway (C1S, C1R, C2, C4), lectin pathway (MBL2, FCN1, MASP1), alternative pathway (CFB, CFD), and the C3 component shared by the three activation pathways were detected in these cells. The components shared by the terminal pathways including C5, C6, C7, C8 and C9 exhibited lower expression, while complement regulators (CFH, CFI, CD55/DAF, CD46/MCP, CD59, C4BPB, PROS1/Protein S) or receptors (CD93/C1QR1, CR1), particularly membrane-bound proteins, such as DAF, MCP and CD59, which inhibit complement activation and the formation of the membrane attack complex, showed relatively high expression.

**Conclusion:**

These results showed that all four types of intrinsic renal cells expressed multiple complement components associated with the classical, lectin, and alternative pathways. In non-diseased kidney tissue, complement regulatory molecules involved in the control of complement activation showed relatively higher expression, whereas components of the terminal complement pathway were expressed at relatively lower levels, suggesting that renal intrinsic cells maintain a locally poised but tightly regulated complement system.

## 1 Introduction

The complement system, comprising more than 30 proteins, can be divided into three parts: intrinsic components, complement regulatory proteins, and complement receptors, and is the central to the innate and adaptive immunity (1, 2). The malfunction of complement system, either deficiency or excessive activation, can disrupt immune homeostasis and contribute to the development of a wide range of diseases (3, 4). The kidney seems to be exceptionally susceptible to complement-mediated injury (5, 6). Accumulating evidence has proven the role of complement components not only in complement-mediated renal disorders, such as C3 glomerulopathy (7) and hemolytic uremic syndrome (8), but also in acute kidney injury (AKI) (9), IgA nephropathy (IgAN) (10), membranous nephropathy (MN) (11), diabetic nephropathy (DN) (12), and lupus nephritis (LN) (13).

Although it is generally believed that complement components deposited in kidneys originate from the hepatocytes, which is considered for synthesis of 90% of complement components, or from the local immune cells such as monocytes and B lymphocytes, which can also synthesize complement under inflammation or immune activation and participate in local immune responses (1, 2). However, increasing evidence indicates that the kidney itself is capable of synthesizing many components of the complement activation cascade (14, 15). Since the initial report by Feucht et al. (16) in 1989, numerous studies have demonstrated that renal resident cells, including glomerular mesangial cells (GMCs), glomerular endothelial cells (GECs), podocytes, and proximal tubular epithelial cells (PTECs), can express various complement components. Nevertheless, most previous studies of complement expression in renal resident cells relied primarily on *in situ* hybridization to assess gene transcription at the tissue level, providing limited cell-type-specific resolution and lacking a systematic characterization of complement expression across different renal cell populations (14).

More recently, Li et al. (15) analyzed single-cell RNA-sequencing data and demonstrated that multiple complement-related genes were expressed in normal mouse kidneys, with complement receptor genes showing the broadest cellular distribution and GMCs exhibiting a particularly prominent complement transcriptional profile. Compared with the mouse, human kidney data showed similar expression patterns but more abundant complement gene expression. Compared to their research, this is the first study to integrate primary cell culture, public single-cell RNA sequencing datasets, 10×Genomics single-cell RNA sequencing, and tissue-level immunohistochemistry to cover the full chain of “transcription-protein-tissue localization” of complement components.

Therefore, this study aimed to systematically characterize the transcript and protein expression profiles of complement components across four types of intrinsic renal cell (GECs, GMCs, podocytes, and PTECs) and to delineate their cell type-specific distribution in non-diseased renal tissue.

## 2 Materials and methods

### 2.1 Cell culture

The primary GMCs and primary GECs (both ScienCell Research Laboratories, Carlsbad, CA, USA), the immortalized human PTEC line (HK-2, American Type Culture Collection) were cultured according to the manufacturers’ instruction in a humidified incubator at 37 °C with 5% CO_2_. The conditionally immortalized human podocyte line was kindly provided by Professor Moin A. Saleem (Children’s Renal Unit and Academic Renal Unit, University of Bristol, Bristol, UK). Podocytes were cultured under growth-permissive conditions at 33 °C and subsequently differentiated at 37 °C for 10–14 days before use in experiments. Primary GECs and GMCs were used at passages 3-5. The immortalized podocyte line was used between passages 7 and 10. HK-2 cells were used between passages 7 and 10 to minimize phenotypic drift associated with prolonged culture.

### 2.2 RNA extraction and complementary DNA synthesis

Total RNA was extracted from cultured cells using the RaPure Total RNA Kit (Magen Biotech Co., Ltd., Guangzhou, China) according to the manufacturer’s instructions. The concentration and purity of RNA were assessed using a NanoDrop spectrophotometer (Thermo Fisher Scientific, Inc., Waltham, MA, USA). First-strand cDNA was synthesized from 2 μg of total RNA using the RevertAid First Strand cDNA Synthesis Kit (Thermo Fisher Scientific, Inc.) according to the manufacturer’s protocol. Conventional reverse transcription polymerase chain reaction (RT-PCR) was performed to determine the presence or absence of transcripts from 30 complement genes in the cultured cells. The primers used for RT-PCR were designed using NCBI Primer-BLAST. The sequences and product lengths of the primers used are listed in **Supplementary Table 1**, whereas the reaction system and thermocycling conditions are detailed in **Supplementary Tables 2** and **3**, respectively. Amplification products were separated by electrophoresis on 1% agarose gels and identified under ultraviolet light according to the position of the strip. Peripheral blood mononuclear cells (PBMCs) isolated from a healthy 31-year-old female donor were used as positive controls for complement transcript detection. Conventional RT-PCR was used as a qualitative detection assay to determine whether complement transcripts are present or absent in each cell type. Band intensities were not used for quantitative comparisons of expression levels, as this would be affected by differences in primer efficiency, amplification length, and cycling conditions. All RT-PCR experiments were performed in at least three independent biological replicates.

### 2.3 Immunofluorescence

HK-2 cells, GMCs, GECs, and podocytes were cultured in confocal dishes, and fixed with 4% paraformaldehyde for 30 min at room temperature. After washing with phosphate-buffered saline (PBS), the cells were permeabilized with Triton X-100 for 15 min at room temperature and blocked with 5% fetal bovine serum/PBS for 1 h. The cells were subsequently incubated with the corresponding primary antibodies overnight at 4 °C. For each primary antibody, parallel staining with a rabbit IgG isotype control at an equivalent molar concentration was performed to assess nonspecific immunofluorescence signals. After washing with PBS, the cells were incubated with the appropriate secondary antibodies for 1 h at room temperature. Cell nuclei were stained with 4′,6-diamidino-2-phenylindole (DAPI). For immunofluorescence co-staining of podocytes and GECs, following complement protein immunostaining, the cells were incubated with directly fluorophore-conjugated antibodies against WT1 or CD31, respectively, for 1 h at room temperature in the dark. After washing three times with PBS, the nuclei were counterstained with DAPI. Fluorescence images were acquired on a Zeiss LSM 880 using identical laser power, gain, pinhole, and pixel dwell time settings across all experimental groups. All immunofluorescence staining experiments were performed in at least three independent biological replicates.

### 2.4 Antibodies used for immunofluorescence analysis

The following antibodies and staining reagents were used for immunofluorescence analysis: rabbit monoclonal antibodies against human CD55 (Abcam, Cat. No. ab243231; 1:100), CD46 (Abcam, Cat. No. ab273583; 1:250), CD59 (Abcam, Cat. No. ab253239; 1:200), C3 (Abcam, Cat. No. ab181147; 1:100), and C4BPB (Abcam, Cat. No. ab199430; 1:250); a rabbit IgG isotype control antibody (Absin, Cat. No. abs20035; 1:200); a mouse monoclonal antibody against human PDGFRB (Abcam, Cat. No. ab69506; 1:200); biotinylated lotus tetragonolobus lectin (LTL; Vector Laboratories, Cat. No. B13252-2; 1:200); a fluorescein isothiocyanate-conjugated mouse monoclonal antibody against human CD31 (BioLegend, Cat. No. 303103; 1:200); an Alexa Fluor^®^ 647-conjugated recombinant rabbit monoclonal antibody against WT1 (Abcam, Cat. No. ab202639; 1:200); an Alexa Fluor™ 488-conjugated cross-adsorbed goat anti-rabbit IgG (H+L) secondary antibody (Thermo Fisher Scientific, Cat. No. A-11008; 1:300); an Alexa Fluor^®^ 594-conjugated affinity-purified goat anti-mouse IgG (H+L) secondary antibody (ZSGB-BIO, Cat. No. ZF-0513; 1:300); and allophycocyanin-conjugated streptavidin (BioLegend, Cat. No. 405207; 1:200).

### 2.5 Immunohistochemical data from the Human Protein Atlas

IHC staining images of complement proteins in normal human kidney tissue were obtained from the Human Protein Atlas (www.proteinatlas.org). The source of normal kidney tissue in the Human Protein Atlas is surgical nephrectomy specimens or cadaveric donor kidneys with histologically confirmed normal renal parenchyma. Details on normal tissue source and antibodies used for IHC staining were provided in **Supplementary Tables 4** and **5**, respectively.

### 2.6 Analysis of the publicly available single-cell RNA-sequencing dataset GSE160048

The publicly available single-cell RNA-sequencing dataset GSE160048 included kidney biopsy samples from eight healthy living kidney donors in Sweden, comprising three men and five women, with a mean age of 48.2 ± 12.0 years. All donors were excluded from hypertension, diabetes mellitus (DM), or other diseases potentially affecting kidney function. All participants were volunteers undergoing kidney donation for recipients with end-stage kidney disease, and kidney biopsy samples were collected from these donors without additional selection based on clinical or molecular characteristics (16). The processing and quality-control procedures for the raw sequencing data have been described in detail by He et al. (16). Data were accessed via the publicly available Gene Expression Omnibus (GEO) database at https://www.ncbi.nlm.nih.gov/geo/query/acc.cgi?acc=GSE160048. No batch correction was applied as the data were already processed as a unified dataset by the original investigators (16).

### 2.7 Patient samples and ethics statement for 10×Genomics single-cell RNA sequencing

This study was conducted in accordance with the principles of the Helsinki Declaration and was approved by the Ethical Committee of Peking University Third Hospital (approval no. S2018207). Written informed consent was obtained from each participant, explicitly authorizing the collection and use of kidney tissue for single-cell RNA sequencing and complement-related research. Renal cortical tissues were obtained from two male patients who underwent renal nephrectomy for urologic malignancies. Tissue samples were collected from histologically normal renal parenchyma located at a distance from the tumor. Participants were excluded from chronic kidney disease (CKD), hepatitis B or C virus infection, autoimmune diseases, hypertension, DM, syphilis, or acquired immunodeficiency syndrome. Detailed clinical characteristics of participants are provided in **Supplementary Table 6**.

### 2.8 10×Genomics library preparation, sequencing, and data processing

Glomeruli were enriched as previously described (17). Briefly, renal cortical tissues were cut into small pieces and sequentially passed through an 80-mesh sieve and a 140-mesh sieve. The glomerular fraction retained on the 140-mesh sieve was collected and digested with collagenase I to generate a single-cell suspension. The cell concentration was adjusted to 1,000 cells/μL, and approximately 7,000 cells in a total volume of 7 μL were loaded for cDNA library construction and single-cell RNA sequencing in accordance with the manufacturer’s standard protocol. The Cell Ranger software pipeline (version 6.1.2) was used to demultiplex cellular barcodes and map reads to the genome. Firstly, We filtered cells which were predicted as double cells by using DoubletFinder software. Next, Seurat object was generated by Seurat package (version 4.1.1) with R software (version 4.0.3) following these criteria: (1) min.cells = 3; (2) 200 < nFeature_RNA< 6,000; (3) 500 < nCount_RNA< 50,000; (4) percent.mt< 20%. In other words, genes expressed in at least 3 cells and cells detected gene number ranged from 200 to 6,000 were kept for further analysis. Low-quality cells were filtered if ≥20% unique molecular identifiers (UMIs) derived from the mitochondrial genome. The key quality control metrics of the 10×Genomics single-cell sequencing datasets were shown in **Supplementary Table 7**. The raw counts were normalized using 10,000 as the scale factor per cell and log (count+1) transformed. The top 2,000 highly variable genes were identified using the FindVariableFeatures function. The normalized data were then scaled, and principal component analysis (PCA) was performed using the RunPCA function with 30 principal components calculated. To mitigate donor-specific batch effects, the PCA embeddings were integrated using the RunHarmony function implemented in the harmony R package (version 1.2.3). The first 30 Harmony-corrected dimensions were used for downstream analyses. Dimensionality reduction was performed using RunTSNE (perplexity = 30). Unsupervised clustering was performed using FindNeighbors and FindClusters (resolution = 0.4). Cell clusters were subsequently classified and annotated based on the selected classic markers (**Supplementary Table 8**).

## 3 Results

### 3.1 Complement gene expression in cultured human intrinsic cells by RT-PCR

**Figure 1:**
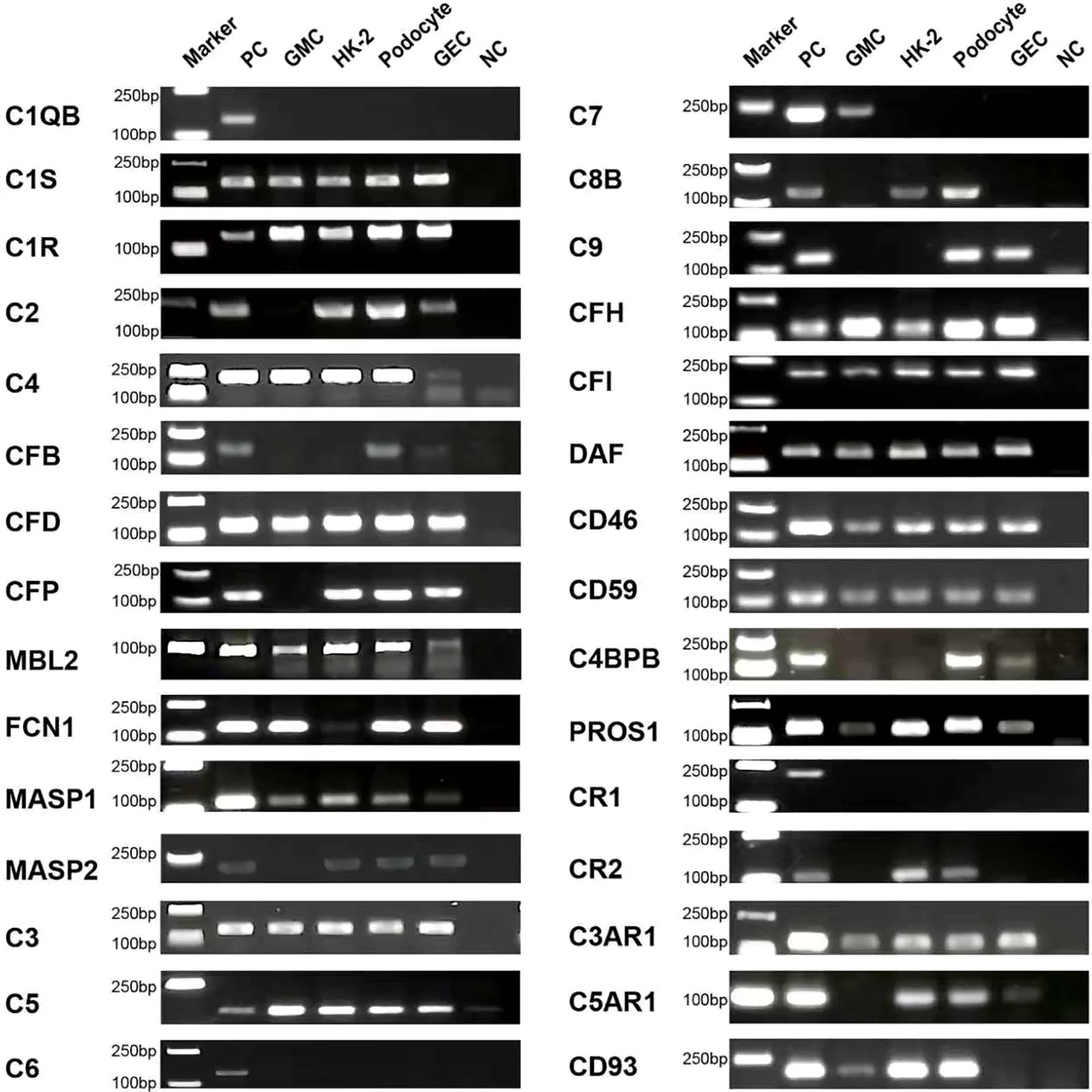
Expression of complement genes in cultured human GECs, GMCs, podocytes, and HK-2 cells. Various complement genes were detected in cultured human GECs, GMCs, podocytes and HK-2 cells. Fifteen of the 30 genes examined were detected in all four renal intrinsic cell types, including C1S, C1R, C4, CFD, MBL2, MASP1, MASP2, C3, C5, CFI, CD55, CD46, CD59, PROS1, and C3AR1. Components of the terminal complement pathway, including C5, C6, C7, C8, and C9, were detected less consistently across the four cell types, whereas complement regulatory genes, including CFH, CFI, CFP, CD55, CD46, CD59, C4BPB, and PROS1, and complement receptor genes, including CD93, C3AR1, and C5AR1, were more broadly detected. A BM2000 DNA marker spanning 100 bp to 2 kb was used as the molecular size ladder, with bands corresponding to 100, 250, 500, 750, 1,000, and 2,000 bp indicated in the figure. PBMCs were used as the positive control (PC) for complement transcript detection, whereas nuclease-free water was used as the negative control (NC).

Conventional RT-PCR was performed to examine the presence of complement-related transcripts in primary human GECs, GMCs, an immortalized human podocyte cell line, and the human PTEC cell line HK-2. The expression of housekeeping genes in the four types of renal intrinsic cells is shown in **Supplementary Figure 1**, whereas the expression of cell type-specific marker genes is presented in **Supplementary Figure 2**. As shown in **Figure 1**, transcripts encoding complement components involved in the initiation of the classical pathway (C1S, C1R, C2, and C4), lectin pathway (MBL2, FCN1, and MASP1), and alternative pathway (CFB and CFD), as well as C3, a central component shared by all three activation pathways, were detected in the cultured renal cells. Among the 30 complement genes examined, 18 were detected in GMCs, 22 in HK-2 cells, 26 in podocytes, and 23 in GECs. Fifteen complement genes were detectable in all four types of renal intrinsic cells, including complement components unique to the classical pathway (C1S, C1R, and C4), alternative pathway (CFD), and lectin pathway (MBL2 and MASP1); components shared by the three activation pathways (C3 and C5); complement regulators (CFH, CFI, CD55/DAF, CD46/MCP, CD59, and PROS1/protein S); and complement receptor (C3AR1).

### 3.2 Immunofluorescence detection of complement proteins in cultured human GECs, GMCs, podocytes, and HK-2 cells

**Figure 2:**
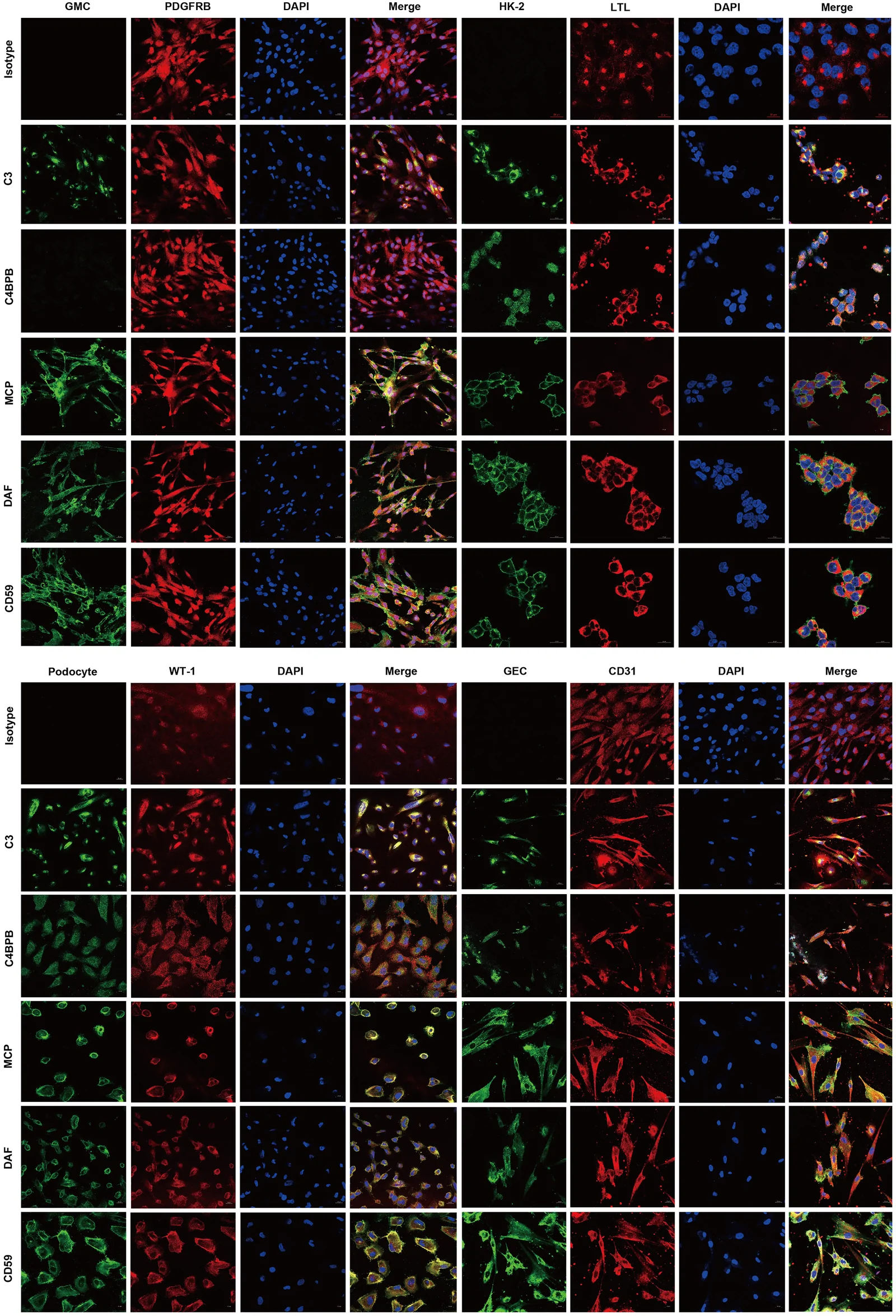
Immunofluorescence detection of complement proteins in cultured human GECs, GMCs, podocytes, and HK-2 cells. C3, a central component shared by all three complement activation pathways, was detected predominantly in the cytoplasm of GECs, GMCs, podocytes, and HK-2 cells. C4BPB was detected mainly in the cytoplasm of GECs and podocytes. These protein-level findings were concordant with the corresponding transcript detection results obtained by RT-PCR. DAF, MCP, and CD59 were localized predominantly to the plasma membrane in all four cell types. Complement protein immunoreactivity is shown in green, cell type-specific markers (including PDGFRB for GMCs, LTL for HK-2 cells, WT-1 for podocytes, and CD31 for GECs) are displayed in red, and nuclei counterstained with DAPI are shown in blue. Rabbit IgG served as the isotype control. Scale bar, 50 µm.

To further validate the expression and subcellular localization of complement proteins in the four types of cultured human renal cells, immunofluorescence staining was performed for C3, a central component shared by all three complement activation pathways, and four complement regulatory proteins, including C4BPB, DAF, MCP, and CD59. As shown in **Figure 2**, C3, was detected predominantly in the cytoplasm of GECs, GMCs, podocytes and HK-2 cells. In contrast, MCP, DAF, and CD59 were localized mainly to the plasma membrane in all four cell types. C4BPB, a regulatory component that inhibits the classical and lectin complement pathways, was detected predominantly in the cytoplasm of GECs and podocytes. These protein-level findings were concordant with the corresponding transcript detection results obtained by RT-PCR.

### 3.3 Complement protein expression in normal human glomeruli and renal tubular epithelial cells based on IHC database analysis

**Figure 3:**
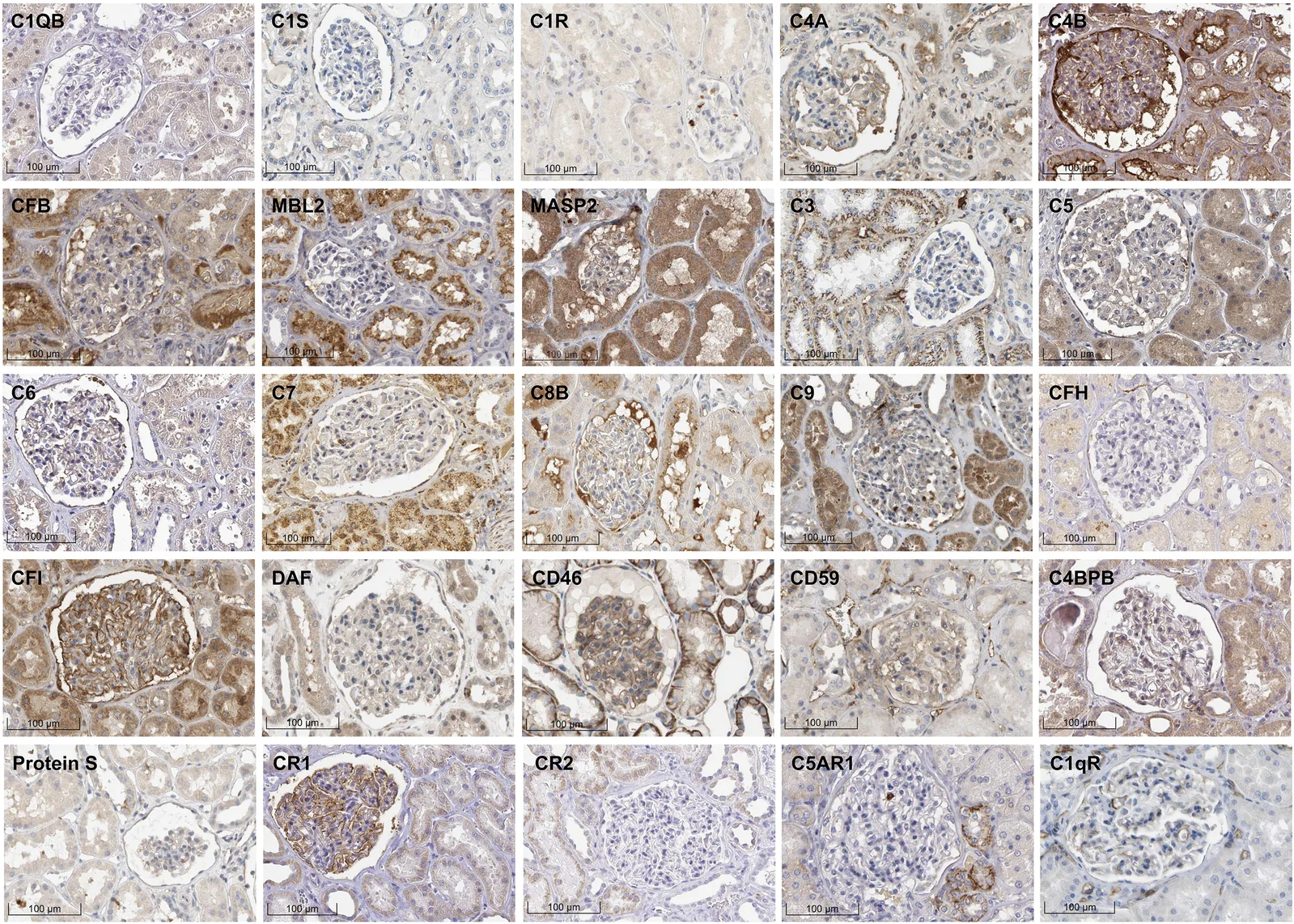
Complement protein expression in normal human glomeruli and renal tubular epithelial cells. The IHC images of normal human kidney tissue were reproduced from the Human Protein Atlas (www.proteinatlas.org) under the Creative Commons Attribution-ShareAlike 3.0 International License (CC BY-SA 3.0). 28 types of complement proteins were retrieved and 24 were detected in normal human renal tissues both in glomeruli and in tubular epithelial cells. Comparison with the transcript detection results in cultured renal intrinsic cells revealed broadly concordant expression patterns. Complement genes detected in glomerular intrinsic cells, including DAF, MCP, CD59, protein S, CR1, and C1QR etc, corresponded to evident protein immunoreactivity within the glomeruli of normal human kidney tissue. Similarly, complement-related genes detected in PTECs showed corresponding protein immunoreactivity in the renal tubular compartment.

Immunohistochemical staining data for 28 complement proteins were retrieved from the Human Protein Atlas (**Figure 3**). Among these proteins, 24 were detectable in normal human kidney tissue, with immunoreactivity observed in both glomeruli and renal tubular epithelial cells. In contrast, four complement proteins, including C2, FCN1, MASP1, and C3AR1 were not detected in in either compartment (**Supplementary Figure 3**). Comparison of the IHC findings with the transcript detection results obtained in cultured renal cells revealed broadly concordant expression patterns. Complement genes detected in glomerular intrinsic cells, including C1S, C1R, C7, CD55, CD46, CD59, PROS1, CR1, and CD93/C1QR1, corresponded to detectable, and in several cases prominent, protein immunoreactivity within the glomeruli of normal human kidney tissue. Similarly, CFI, CD46, CD59, and PROS1, which were detected at the transcript level in PTECs, exhibited evident immunoreactivity in the renal tubular compartment. Collectively, these findings provided tissue-level support for the complement expression profiles observed in cultured renal intrinsic cells.

### 3.4 Complement gene expression in normal human renal intrinsic cells based on public single-cell RNA-sequencing data

**Figure 4:**
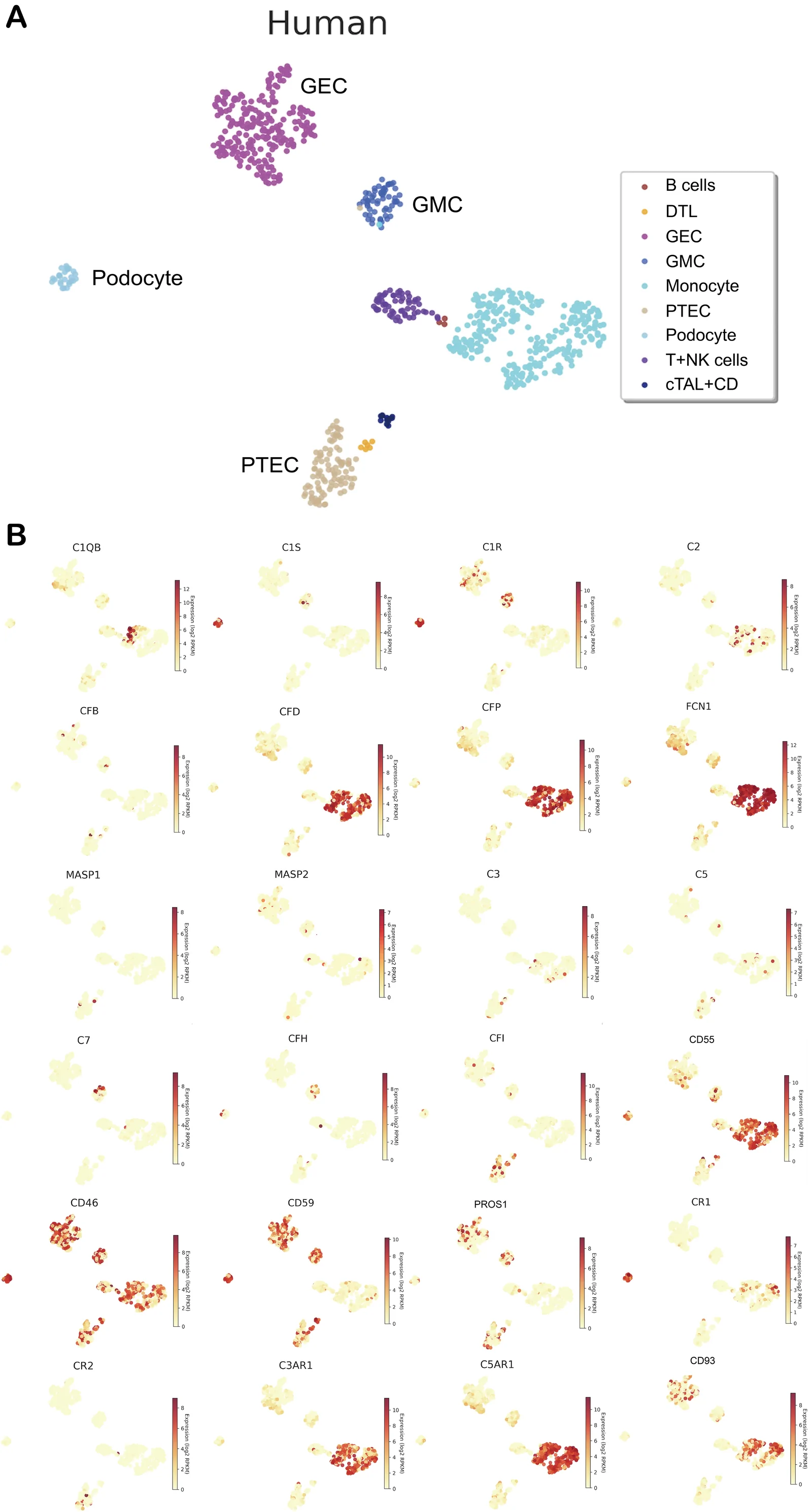
Complement expression in normal human intrinsic renal cells by single-cell RNA sequencing dataset analysis. **(A)** Unsupervised clustering analysis of the single cells identified nine distinct cell clusters. **(B)** Complement genes identified by single cell RNA sequencing database analysis in GECs, GMCs, podocytes, and PTECs. The color intensity of dots represents the expression level. The color scale is defined by log2. GEC, glomerular endothelial cell;GMC, glomerular mesangial cell; PTEC, proximal tubule epithelial cell; cTAL, cortical thick ascending limb; CD, collecting duct; DTL, descending thin limb.

Single-cell complement gene-expression data from the GSE160048 dataset, comprising normal kidney biopsy samples from eight healthy living kidney donors, were obtained from the Gene Expression Omnibus database. By retrieving the public searchable database (https://www.ncbi.nlm.nih.gov/geo/query/acc.cgi?acc=GSE160048), nine major cell clusters of cells were identified. Clusters expressing PECAM1, PDGFRB, NPHS1, and PCK1 were annotated as GECs, GMCs, podocytes, and PTECs, respectively. Uniform manifold approximation and projection (UMAP) demonstrated clear separation of the annotated cell populations, reflecting distinct cell type-specific transcriptional profiles (**Figure 4A**). Feature plots further revealed heterogeneous and cell type-specific expression patterns of complement genes (**Figure 4B**). A total of 24 complement-related genes were detected across the four renal intrinsic cell populations, including C1Q, C1S, C1R, C2, C3, C5, C7, MASP1, MASP2, CFB, CFD, CFP, FCN1, CFH, CFI, CD55, CD46, CD59, PROS1, CR1, CR2, C3AR1, C5AR1, and CD93. Components of the classical pathway, including C1R and C1S, were predominantly enriched in podocytes, whereas C1QB was mainly restricted to infiltrating monocytes. Among alternative pathway genes, CFB showed strong and widespread expression in PTECs, while CFD and CFP were primarily detected in monocytes with relatively weaker expression in intrinsic renal cells. The central complement component C3 was preferentially expressed in PTECs, whereas C7 was enriched in GMCs. Complement regulatory molecules displayed distinct distributions, with CFI predominantly expressed in PTECs, CFH predominantly expressed in GMCs, whereas CD46 and CD59 were broadly expressed in GECs and podocytes. Complement receptors C3AR1 and C5AR1 were almost exclusively localized to monocytes, whereas CR1 was mainly detected in podocytes and CD93 was selectively enriched in GECs.

### 3.5 Complement gene transcripts were detected in normal human intrinsic renal cells by 10×Genomics single-cell RNA sequencing

**Figure 5:**
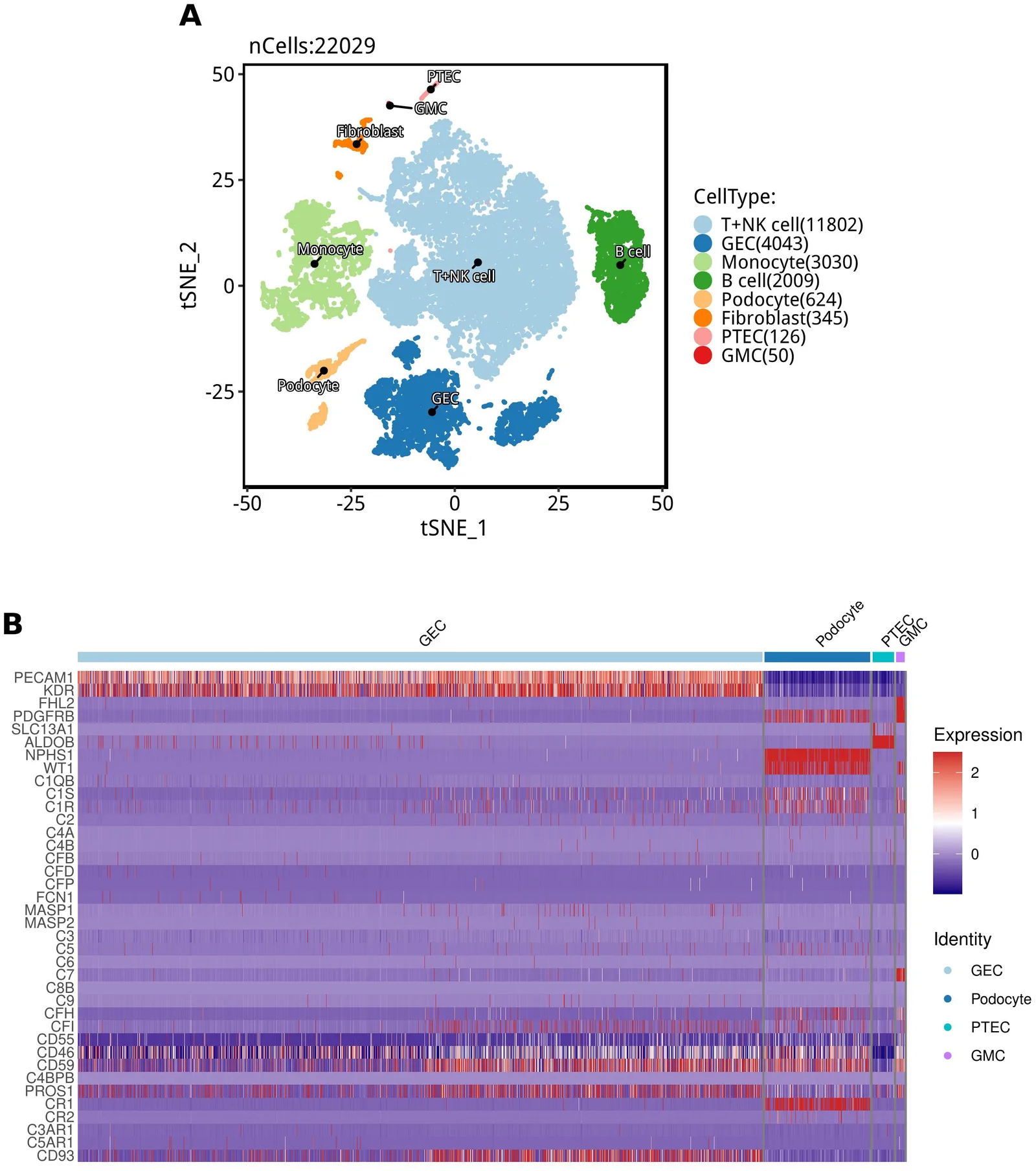
Complement expression of human intrinsic renal cells by 10×Genomics single-cell RNA sequencing. **(A)** The plot shows a two-dimensional representation (tSNE: t-distributed stochastic neighbor embedding) of global relationships of all the sequenced single cells. **(B)** Heatmap of the expression of 30 complement genes in four types of intrinsic renal cells. Color scheme represents the z-score distribution from −1.0 **(blue)** to 2.5 (red). The z-score for each gene was calculated across the four renal intrinsic cell types as z = (x - mean)/standard deviation (SD), where x is the mean log-normalized expression in a given cell type and mean and SD are computed across all four cell types for that gene. GEC, glomerular endothelial cell; GMC, glomerular mesangial cell; PTEC, proximal tubule epithelial cell; DTC, distal tubular cells.

Single-cell sequencing is the ultimate method for examining cellular expression. After applying the quality control filters, we analyzed a total of 22,029 cells with a median of 2,079 genes per cell. As shown in **Figure 5A**, unsupervised clustering analysis of single cells identified ten distinct cell clusters based on known cell genes markers which are shown in **Supplementary Table 8**. The clusters with PECAM1^+^/KDR^+^, FHL2^+^/PDGFRB^+^, NPHS1^+^/WT1^+^, and SLC13A1^+^/ALDOB^+^ are assigned as GECs, GMCs, podocytes, and PTECs, respectively. The expression of cell-specific markers in each cell type are shown in **Supplementary Figure 4**. As shown in **Figures 5B**, single-cell transcriptomic heatmap analysis revealed distinct cell type-specific expression patterns of complement genes across GECs, podocytes, PTECs, and GMCs. Most genes encoding the core components of the complement cascade, including classical pathway components (C1QB, C2, C4A, and C4B), lectin pathway-associated genes (FCN1, MASP1, and MASP2), alternative pathway components (CFB, CFD, and CFP), and central or terminal pathway components (C3, C5, C6, C7, C8B, and C9), exhibited generally low or sporadic expression. Nevertheless, marked cellular heterogeneity was observed. Podocytes showed relatively prominent expression of C1R and C1S, together with strong enrichment of the complement receptor CR1 and detectable expression of the regulatory genes CFH, CFI, and CD59. GECs preferentially expressed complement-regulatory or complement-associated genes, including PROS1, CFI, CD46, CD59, and CD93, with CD93 displaying a highly GEC-restricted expression pattern. In contrast, PTECs exhibited limited transcription of most complement-related genes, with only scattered expression of genes such as CFB, MASP1, CFI, and PROS1. GMCs were characterized by relatively increased expression of C7 and CD59, accompanied by variable expression of C1R, CFH, CD46, CD55, and PROS1. The anaphylatoxin receptors C3AR1 and C5AR1 were expressed at low levels across all four renal parenchymal cell populations. As shown in **Supplementary Figure 5**, the dot plot further complemented the heatmap by simultaneously depicting the average expression level and the proportion of cells expressing each complement gene. Podocytes exhibited the broadest complement-associated transcriptional profile, characterized by a relatively high prevalence and expression of C1S, C1R, CR1, CD46, and CD59. GMCs displayed a more selective pattern, with prominent C7 expression accompanied by several complement regulatory genes, whereas GECs predominantly expressed complement-protective and regulatory molecules, including CD46, CD59, PROS1, and CD93. In contrast, most complement-related genes were detected in only a small proportion of PTECs.

## 4 Discussion

In this study, we systematically characterized the expression of complement components across four major intrinsic renal cell types, including GMCs, GECs, podocytes, and PTECs. Transcript- and protein-level analyses provided descriptive evidence that these cells express a broad range of complement components associated with the classical, lectin, and alternative pathways. In non-diseased renal tissue, complement regulatory molecules associated with the control of C3 activation and membrane attack complex (MAC) formation generally exhibited relatively higher expression than components of the terminal complement pathway. Collectively, these findings delineate a cell type-specific complement expression landscape within the kidney and provide a descriptive framework for future functional studies of locally produced complement components and regulators.

Local production of complement was initially discovered in renal diseases. As early as in 1988, Passwell found that the kidney of mice with LN expressed increased amounts of RNA message for C4, C2, C3, and CFB (18). One year later, Feucht described the isolation of gene transcripts for C4 from the cortex of normal human kidney (19). Brooimans et al. (20) reported the synthesis of complement by PTECs grown from normal human kidney and Witte et al. (21) confirmed that the renal tubule was a prominent site for local complement gene expression in normal and diseased kidneys. Sack found that cultured human GMCs and epithelial cells were shown to be able to secrete a variety of classic and alternative pathway complement components (22–24). Montinaro reported the biosynthesis of C3 by human GMCs (25). Recently, Li X et al. (15) reported complement gene expression patterns in mouse and human kidneys using publicly available single-cell RNA sequencing datasets and found that complement genes were widely expressed in kidney cells with GMC as the most abundant cell type. The present study extends this knowledge by applying a multi-platform validation strategy that uniquely integrates five complementary approaches: RT-PCR and immunofluorescence in cultured human renal intrinsic cells, IHC data from the Human Protein Atlas, reanalysis of the public human kidney single-cell RNA sequencing dataset, and an original 10×Genomics single-cell RNA sequencing experiment performed on non-diseased human kidney tissue. To our knowledge, this is the first study to simultaneously apply this level of multi-platform cross-validation across all four major human renal intrinsic cell types in a single investigation. Additionally, our study highlighted species-specific features of complement expression in different types of human renal intrinsic cells, which strengthens the transforming potential for cell-type-specific complement-targeted therapies. For example, the relatively high baseline expression of CFI in PTECs raises the possibility that these cells could serve as a therapeutic target for complement-regulatory gene delivery or CFI supplementation, thereby enhancing local complement control and potentially limiting complement-mediated tubular injury in AKI and CKD.

The complement expression profiles in non-diseased renal tissue described in this study likely represent a regulatory steady state that can be substantially perturbed under inflammatory conditions. Previous studies have demonstrated that pro-inflammatory cytokines including TNF-α, IL-1α and TGF-β can upregulate complement activating components (such as C3) and downregulate regulatory proteins in renal cells, potentially shifting the complement balance toward a pro-inflammatory, pro-injury phenotype (26–28). The complement expression profiles defined in the current study thus provide an important reference baseline against which complement dysregulation in inflammatory kidney diseases, including IgAN, LN and DN, and can be compared in future studies.

It was suggested that there are regional differences in complement mRNA expression within the kidney (29). Zhou W et al. (14) indicated that the main components of complement synthesized in the liver (C2, C4, C3, and CFB) are more strongly detected in the renal cortex consisting mainly of tubules, whereas some components that are primarily produced at extrahepatic sites (for example, C1Q and CFD) are detected strongly in glomeruli, supposing that certain factors have a specialized function in the glomerulus. In addition to the complement activation pathway components, a number of complement regulatory proteins particularly membrane-bound proteins, are synthesized within the kidney (30). These include DAF, MCP, CR1, and CD59. DAF, MCP, and CR1 inhibit complement activation at the level of the C3 and C5 converting enzyme complexes, while CD59 prevents the formation of the MAC. It has been found that complement regulatory proteins are widely expressed in glomeruli and renal tubules with the level of expression varying with the anatomical segment or cell type of the kidney (31). The renal tubule is relatively deficient in DAF and CD59, helping to explain the vulnerability of the renal tubule to complement attack (32). Expression of these molecules is up-regulated during the activation of complement (33), as occurs in a number of kidney diseases (34, 35). Not surprisingly, over-expression of complement regulatory protein in mice markedly ameliorates nephrotoxic serum nephritis (36). Li X reported that complement genes were widely expressed in kidney cells with the receptor genes most widely expressed (15). In our study, complement regulatory molecules associated with the control of C3 activation and MAC formation showed relatively higher expression in non-diseased renal tissue. Together with the expression of multiple complement components, this pattern is consistent with the presence of a locally organized complement-related expression network within the kidney. However, whether these expression patterns translate into functional modulation of complement activation on renal structures requires direct experimental validation.

At single-cell resolution, our findings delineate a compartmentalized complement transcriptional landscape across resident renal cell populations in non-diseased renal tissue. GECs, which line in the luminal surface of glomerular capillaries and are in direct, continuous contact with circulating complement proteins, exhibit the highest expression of complement inhibitory regulators including DAF, MCP, CD59, and protein S. This predominance of regulatory over activating complement genes in GECs is consistent with a constitutive protective mechanism to prevent inadvertent complement activation on the endothelial surface, a principle well-established in the broader literature on vascular endothelial complement biology (37). In contrast, podocytes exhibited a broader complement-related repertoire encompassing classical-pathway serine proteases (C1R and C1S) and the complement receptor CR1. CR1 is selectively expressed on podocytes and can provide factor I cofactor activity for the degradation of deposited C3b, indicating a potential role in the local clearance and inactivation of complement-opsonized material (38). Moreover, reduced or proteolytically cleaved podocyte CR1 has been associated with terminal complement complex deposition and more severe glomerular injury in LN, supporting the concept that disruption of this regulatory interface may increase podocyte vulnerability to complement-mediated damage (39). Thus, the coexistence of complement-initiating, sensing, and regulatory transcripts in podocytes may represent a tightly controlled surveillance system that permits local complement handling while limiting MAC-mediated injury. Although PTECs showed relatively weak complement transcription in the present dataset, this should not be interpreted as evidence of biological inactivity. Human PTECs can synthesize functional C3, constitutively express C4, and induce C3 and CFB expression in response to inflammatory stimuli or tubulointerstitial injury (20, 40, 41). Therefore, the low expression observed here may reflect a relatively quiescent basal state, whereas inflammatory or metabolic stress could convert PTECs into an important source of locally generated complement components. Finally, the selective enrichment of C7 together with the membrane regulator CD59 in GMCs suggests a dual program in which GMCs may contribute to terminal complement pathway assembly while simultaneously protecting themselves from autologous MAC injury. Experimental studies have demonstrated functional complement regulation by CD59 in glomerular resident cells, and single-cell reanalysis has identified C7 as a mesangial-enriched gene in diabetic kidney disease (42, 43). Collectively, these observations support a model in which each renal cell type maintains a distinct balance between complement production, recognition, amplification, and inhibition. Disturbance of this cell-specific equilibrium may determine whether complement functions as a homeostatic surveillance system or becomes a driver of glomerular and tubulointerstitial injury. Importantly, because transcript abundance does not directly establish protein secretion, proteolytic activation, or functional complement activity, these single-cell findings warrant validation by spatial transcriptomics, immunostaining, proteomic analysis, and assays of complement deposition and activation.

There are discrepancies of complements expression among different studies, which may result from various factors such as different methods and confounders of mRNA-protein dependencies like temporal and spatial contexts (44). At the same time, it seemed that no type of renal intrinsic cell could simultaneously express all complement components needed in one activation pathway, especially those involved in the terminal pathway to form MAC. The consistently low expression of terminal pathway components (C5, C6, C7, C8, and C9) across all four renal intrinsic cell types is a notable finding with important mechanistic implications. These data may suggest that human renal intrinsic cells are unlikely to be autonomous producers of MAC, and that terminal complement activation in the kidney under pathological conditions most probably depends on circulating hepatic complement proteins. This is consistent with the established view that the liver supplies approximately 90% of circulating complement proteins, and that locally produced renal complement contributes primarily to the early and amplification phases of complement activation rather than to terminal pathway assembly (45). Nevertheless, even relatively low local expression of C5 by renal tubular cells may be sufficient to generate C5a, a potent pro-inflammatory anaphylatoxin, in the immediate pericellular environment. This local C5a production could amplify tubular inflammation and contribute to the progression of tubulointerstitial disease, as suggested by prior studies implicating tubular C5a in interstitial nephritis (46–48). Future studies should test the hypothesis that silencing C5 expression in PTECs reduces pericellular C5a generation and downstream inflammatory signaling, and that the high constitutive expression of DAF in GECs actively limits C3 convertase assembly and MAC deposition on the glomerular endothelial surface in non-diseased renal tissue.

Our study has several limitations. First, the PTEC data were obtained from HK-2 cells, an immortalized human PTEC cell line. Although HK-2 cells are widely used as an *in vitro* model of PTECs, immortalization-associated transcriptomic alterations may result in a complement expression profile that differs from that of primary PTECs *in vivo*. Validation in primary human PTEC cultures and, where feasible, ex vivo renal tissue would therefore strengthen the generalizability of these findings. Second, the 10×Genomics single-cell RNA-sequencing datasets were derived from kidney tissues obtained from only two male patients undergoing nephrectomy for renal carcinoma. The limited sample size and restricted donor characteristics precluded assessment of inter-individual variation in complement-related gene expression according to age, sex, genetic background, and other biological factors. Moreover, although histologically non-diseased tissue was analyzed, the possible influence of the adjacent tumor and perioperative conditions cannot be completely excluded. Accordingly, the single-cell findings should be considered preliminary and hypothesis-generating and require validation in larger, demographically diverse, multi-donor cohorts. Third, the present study primarily provides transcript- and protein-expression data and does not establish the functional consequences of the observed expression patterns. Immunofluorescence analysis demonstrated the cellular localization of selected complement-related proteins but did not determine whether these proteins were secreted, biologically active, assembled into functional complement complexes, or capable of modulating complement activation on renal cell surfaces. Functional studies are therefore needed to clarify the biological significance of these findings. For example, siRNA-mediated knockdown of DAF in primary GECs, followed by exposure to normal human serum and quantification of C3b and C5b-9 deposition by flow cytometry, could assess whether reduced DAF expression is associated with increased complement deposition on the glomerular endothelial surface. Similarly, stable overexpression of CD59 in HK-2 cells, followed by a complement-mediated cytotoxicity assay using anti-tubular antibodies and human complement-containing serum, with LDH release as a readout, could evaluate whether increased CD59 expression reduces MAC-associated cellular injury. Such experiments, together with analyses of complement secretion and activation products, will be required to determine whether the expression profiles identified in this study translate into biologically meaningful regulation of local complement activity in the kidney.

## 5 Conclusion

This study provides descriptive evidence that GMCs, GECs, podocytes, and PTECs express complement components associated with the classical, lectin, and alternative pathways. In non-diseased renal tissue, terminal pathway components were expressed at relatively lower levels, whereas complement regulators showed relatively higher expression. These findings delineate the cell type-specific complement expression profile of the kidney and warrant further functional validation.

## Data Availability

The original contributions presented in the study are publicly available. This data can be found here: NCBI SRA, accession number PRJNA1513720.
