# Supplementary material for: Complement expression profiles in human glomerular mesangial cells, endothelial cells, podocytes and proximal tubular epithelial cells
Source: Front Immunol. 2026 Sep 1;17:1881549. doi: 10.3389/fimmu.2026.1881549 (PMC13574829; doi:10.3389/fimmu.2026.1881549)
Supplement: Supplementary file 1 [file Table1.docx]

**Supplementary material**

**Supplementary** **Table 1**. List of primers used in PCR

| Gene | NCBI accession number | Primer Sequence (5’-3’) | Product (bp) |
| --- | --- | --- | --- |
| C1QB | NG_007283.1 | Forward AGGTGAATCGGGAGACTACAA  Reverse CACTGCGGGGCTCATAATTG | 143 |
| C1S | NG_011694.1 | Forward TTTGGCATGGGTTTATGCTGA  Reverse GGGTGAAGTAGAGGTGAATCCC | 140 |
| C1R | NG_062465.1 | Forward CACCTGTGTCACAACTACGTT  Reverse GGCTGGAGATGTAGCCTGATG | 136 |
| C2 | NG_011730.1 | Forward TGGAAAGTCCAATATGGGTGGC  Reverse CTGGTGCAGAGCCTTTGTGT | 214 |
| C4 | NG_011638.1 | Forward GGATCAGTGTTCCTGAGAAACC  Reverse GGACAGAGAGTCCTTTAGCCAT | 207 |
| CFB | NG_008191.1 | Forward GCACTGGAGTACGTGTGTCC  Reverse CCCGTTCTCGAAGTCGTGTG | 168 |
| CFD | NG_007274.1 | Forward GACACCATCGACCACGACC  Reverse GCCACGTCGCAGAGAGTTC | 128 |
| CFP | NG_009893.1 | Forward TGCTCTGCTTCACCCAGTATG  Reverse CCACTACGTTTCTGGTAGGCA | 118 |
| MBL2 | NG_008196.1 | Forward CCCTGTTTCCATCACTCCCTC  Reverse GCAGGTCTTTTGGGCATCC | 92 |
| FCN1 | NG_046982.2 | Forward GGGCAGTGCGGGTAATTCTC  Reverse GAAGCATGACAGTCGGCGTA | 135 |
| MASP1 | NG_029440.1 | Forward CACCGTGGAGCTAAACAATATGT  Reverse GCTTGATCCGAAACCCATCTG | 119 |
| MASP2 | NG_007289.2 | Forward TGCAGCATTGTTGACTGTGG  Reverse TGTTGTGCGGGCTGATAGTC | 243 |
| C3 | NG_009557.1 | Forward GGGGAGTCCCATGTACTCTATC  Reverse GGAAGTCGTGGACAGTAACAG | 125 |
| C5 | NG_007364.1 | Forward ACAGTCATAGAGTCTACAGGTGG  Reverse CCAACTGGTCAAGCGAATCTT | 166 |
| C6 | NG_011582.1 | Forward ATTCGAGGTGGAAGGAGTGAA  Reverse GGGGCAAGCTCAAAGTCAATC | 137 |
| C7 | NG_011692.1 | Forward CAATGGGGATTCTGACTGTGATG  Reverse TCCACTCAGCCTGTAGAAATCTT | 235 |
| C8B | NG_007285.1 | Forward ATTCCTTTGGGTCAAATGCAGT  Reverse GGACCAACTAGACAGCTCACA | 107 |
| C9 | NG_009894.1 | Forward AGCACAGTACACGACCAGTTA  Reverse AAGGATCGCATTGTGACCATT | 110 |
| CFH | NG_007259.1 | Forward CACACAAGATGGATGGTCGC  Reverse GGATGGCAGGCAACGTCTAT | 132 |
| CFI | NG_007569.1 | Forward GGTGAGGTGGACTGCATTACA  Reverse CCTCCCACAATTCGTTTCCTTC | 191 |
| CD55/DAF | NG_007465.1 | Forward TTCCAGTCGGTACTGTTGTGG  Reverse CCCGGATTAGGGCATGATTTCT | 145 |
| CD46/MCP | NG_009296.1 | Forward ACCAACATTTGAAGCTATGGAGC  Reverse GCCATGTATGATTCCGATCACAA | 146 |
| CD59 | NG_008057.1 | Forward TTTTGATGCGTGTCTCATTACCA  Reverse ATTTTCCCTCAAGCGGGTTGT | 106 |
| C4BPB | NG_029386.1 | Forward TCTGCAAAAGTAGGGACTGTGA  Reverse CACGCCCACTAAGTAGTACCT | 125 |
| PROS1/Protein S | NG_009813.1 | Forward TCCTGGTTAGGAAGCGTCGT  Reverse CCGTTTCCGGGTCATTTTCAAA | 132 |
| CR1 | NG_007481.1 | Forward AGAGGGACGAGCTTCGACC  Reverse TCAGGACGGCATTCGTACTTT | 212 |
| CR2 | NG_013006.1 | Forward GGTCCTCGGGATTTCTTGTGG  Reverse GAACAACTGTACCTTATCACGGT | 105 |
| C3AR1 | NG_050736.1 | Forward GTTCGTGTACCGGGAAATCTT  Reverse CAGGCGGCTGAACAATGTTT | 143 |
| C5AR1 | NC_000019.10 | Forward TCCTTCAATTATACCACCCCTGA  Reverse ACGCAGCGTGTTAGAAGTTTTAT | 96 |
| CD93/C1QR1 | NC_000020.11 | Forward CCGGAAGTAACATTGAGGGCT  Reverse TCTGAGTCTCGTCCTTGTCAC | 192 |
| NPHS1/nephrin | NG_013356.2 | Forward GGTGCAGTTTCCCCCAACTA  Reverse AGAAGGAGCTCACGGTTTCG | 293 |
| LRP2 | NG_012634.1 | Forward GTGCAGACCTAAAGGAGCGTT  Reverse ATGCAATGCCCACTTCCACA | 228 |
| PECAM1/CD31 | NG_047009.1 | Forward ACCGTGACGGAATCCTTCTCT  Reverse GCTGGACTCCACTTTGCAC | 246 |
| PDGFRB | NG_023367.2 | Forward CTCTGGCGGCTCTGCTCCTC  Reverse CCTTGCTGCTGATGGCTTCTGG | 276 |
| ACTB/β-actin | NG_007992.1 | Forward GAAGTGTGACGTGGACATCC  Reverse CCGATCCACACGGAGTACTT | 177 |

**Supplementary** **Table 2**. The reaction system of RT-PCR used in this study

|  | Positive  control | Complement genes | Negative control |
| --- | --- | --- | --- |
| Water | 8.6 μL | 8.6 μL | 8.6 μL |
| 2×PCR mix | 10 μL | 10 μL | 10 μL |
| Forward primer (10 μM) | 0.2 μL | 0.2 μL | 0.2 μL |
| Reverse primer (10 μM) | 0.2 μL | 0.2 μL | 0.2 μL |
| cDNA | 1 μL | 1 μL | Water 1 μL |
| Total | 20 μL | 20 μL | 20 μL |

**Supplementary Table 3.** Reaction conditions of RT-PCR used in this study

|  | Denaturation | Annealing | Extension |
| --- | --- | --- | --- |
| Temperature/duration (℃/sec) | 95/180 | 95/30-64/30-72/30 | 72/300 |
| Cycle number | 1 | 35 | 1 |

**Supplementary Table 4.** Details on normal tissue source in Human Protein Atlas

| Antigen | Tissue source | Patient id | Patient age | Patient sex |
| --- | --- | --- | --- | --- |
| C1QB | Normal tissue | 2530 | 41 | Female |
| C1S | Normal tissue | 3229 | 59 | Male |
| C1R | Normal tissue | 924 | 36 | Male |
| C4A | Normal tissue | 2165 | 7 | Male |
| C4B | Normal tissue | 2530 | 41 | Female |
| CFB | Normal tissue | 1933 | 56 | Female |
| MBL2 | Normal tissue | 1933 | 56 | Female |
| MASP2 | Normal tissue | 2530 | 41 | Female |
| C3 | Normal tissue | 1933 | 56 | Female |
| C5 | Normal tissue | 2530 | 41 | Female |
| C6 | Normal tissue | 2530 | 41 | Female |
| C7 | Normal tissue | 1933 | 56 | Female |
| C8B | Normal tissue | 1767 | 16 | Male |
| C9 | Normal tissue | 2530 | 41 | Female |
| CFH | Normal tissue | 2530 | 41 | Female |
| CFI | Normal tissue | 1933 | 56 | Female |
| DAF | Normal tissue | 1933 | 56 | Female |
| MCP | Normal tissue | 1933 | 56 | Female |
| CD59 | Normal tissue | 1933 | 56 | Female |
| C4BPB | Normal tissue | 1859 | 61 | Male |
| Protein S | Normal tissue | 1767 | 16 | Male |
| CR1 | Normal tissue | 2530 | 41 | Female |
| CR2 | Normal tissue | 2530 | 41 | Female |
| C5AR1 | Normal tissue | 1933 | 56 | Female |
| C1QR1 | Normal tissue | 1933 | 56 | Female |
| C2 | Normal tissue | 1933 | 56 | Female |
| FCN1 | Normal tissue | 1263 | 52 | Female |
| MASP1 | Normal tissue | 1933 | 56 | Female |
| C3AR1 | Normal tissue | 1767 | 16 | Male |

**Supplementary Table 5.** Details on antibodies used in Human Protein Atlas

| Antigen | Host | Reactivity | Clonality | Source | Product name | Concentration |
| --- | --- | --- | --- | --- | --- | --- |
| C1QB | Rabbit | Human | pAb | Sigma-Aldrich | HPA052116 | 0.0683 mg/ml |
| C1S | Rabbit | Human | pAb | Sigma-Aldrich | HPA018852 | 0.0575 mg/ml |
| C1R | Rabbit | Human | pAb | Sigma-Aldrich | HPA001251 | 0.0673 mg/ml |
| C4A | Rabbit | Human | pAb | Santa Cruz Biotechnology | CAB009811 | Not known |
| C4B | Rabbit | Human | pAb | Sigma-Aldrich | HPA050103 | 0.0514 mg/ml |
| CFB | Rabbit | Human | pAb | Sigma-Aldrich | HPA001817 | 0.07 mg/ml |
| MBL2 | Rabbit | Human | pAb | Sigma-Aldrich | HPA002027 | 0.12 mg/ml |
| MASP2 | Rabbit | Human | pAb | Sigma-Aldrich | HPA029313 | 0.15 mg/ml |
| C3 | Mouse | Human | mAb | Santa Cruz Biotechnology | CAB004209 | Not known |
| C5 | Rabbit | Human | pAb | Sigma-Aldrich | HPA029339 | 0.0581 mg/ml |
| C6 | Rabbit | Human | pAb | Sigma-Aldrich | HPA043823 | 0.091 mg/ml |
| C7 | Rabbit | Human | pAb | Sigma-Aldrich | HPA001465 | 0.03 mg/ml |
| C8B | Rabbit | Human | pAb | Sigma-Aldrich | HPA023694 | 0.017 mg/ml |
| C9 | Rabbit | Human | pAb | Sigma-Aldrich | HPA029577 | 0.589 mg/ml |
| CFH | Rabbit | Human | pAb | Sigma-Aldrich | HPA038922 | 0.0495 mg/ml |
| CFI | Rabbit | Human | pAb | Sigma-Aldrich | HPA024061 | 0.059 mg/ml |
| DAF | Rabbit | Human | pAb | Santa Cruz Biotechnology | CAB010454 | Not known |
| MCP | Rabbit | Human | pAb | Santa Cruz Biotechnology | CAB010401 | Not known |
| CD59 | Rabbit | Human | pAb | Sigma-Aldrich | HPA026494 | 0.0225 mg/ml |
| C4BPB | Rabbit | Human | pAb | Sigma-Aldrich | HPA051620 | 0.2078 mg/ml |
| Protein S | Rabbit | Human | pAb | Sigma-Aldrich | HPA023974 | 0.055 mg/ml |
| CR1 | Rabbit | Human | pAb | Sigma-Aldrich | HPA042455 | 0.1462 mg/ml |
| CR2 | Rabbit | Human | pAb | Sigma-Aldrich | HPA052942 | 0.1731 mg/ml |
| C5AR1 | Mouse | Human | mAb | Agilent (Formerly DakoCytomation) | CAB002720 | Not known |
| C1QR1 | Rabbit | Human | pAb | Sigma-Aldrich | HPA012368 | 0.02 mg/ml |
| C2 | Mouse | Human | mAb | BioPorto Diagnostics A/S | CAB016775 | Not known |
| FCN1 | Rabbit | Human | pAb | Sigma-Aldrich | HPA000685 | 0.2189 mg/ml |
| MASP1 | Rabbit | Human | pAb | Sigma-Aldrich | HPA001617 | 0.09 mg/ml |
| C3AR1 | Rabbit | Human | pAb | Santa Cruz Biotechnology | CAB009405 | Not known |

**Supplementary** **Table 6.** Clinicopathologic characteristics of patients used for 10×Genomics Chromium system.

| Case | Tumor  type | Tumor  stage | Age/  Sex | BP (mmHg) | Proteinuria (g/d) | Serum creatinine (μmol/L) | eGFR (ml/min/1.73m^2^) | Hematuria (0 to 5+ ) |
| --- | --- | --- | --- | --- | --- | --- | --- | --- |
| Case-1 | Ureteral cancer | T2N0M0 | 48/M | 130/86 | ND | 100 | 80 | 2 |
| Case-2 | renal pelvis cancer | T3N0M0 | 57/M | 122/82 | ND | 98 | 74 | 2 |

Abbreviations: M, male; BP: blood pressure; ND, not detected; eGFR: estimated glomerular filtration rate.

**Supplementary Table 7.** The key quality control metrics of the 10×Genomics single cell sequencing datasets

|  | Case-1 | Case-2 |
| --- | --- | --- |
| Estimated number of cells | 5,241 | 16,788 |
| Mean reads per cell | 69,278 | 35,346 |
| Median genes per cell | 1,712 | 2,208 |
| Valid barcodes | 89.97% | 83.40% |
| Sequencing saturation | 89.84% | 50.0% |
| Mapped to genome | 96.10% | 91.8% |

Case-1: 48-year old male patient with ureteral cancer; case-2: 57-year old male patient with renal pelvis cancer.

**Supplementary Table 8.** Cell-cluster specific marker genes used for the 10×Genomics single cell sequencing datasets

| **Cell types** | **Marker genes** |
| --- | --- |
| T+NK cell | CD3D, CD3E, KLRD1, NKG7, IL7R |
| GEC | PECAM1, KDR, VWF, SLC14A1 |
| Monocyte | LYZ, CD14, FCN1 |
| B cell | MS4A1, CD19, CD79A |
| Podocyte | NPHS1, NPHS2, WT1, PTPRO |
| Fibroblast | COL3A1, COL1A2 |
| PTEC | SLC13A1, ALDOB, LRP2, CUBN |
| GMC | PDGFRB, FHL2, GATA3 |

Abbreviations: NK: natural killer; GEC: glomerular endothelial cell; PTEC: proximal tubular epithelial cell; GMC: glomerular mesangial cell.

**Supplementary Figure 1.** The transcription of β-actin in PBMCs, GMCs, HK-2 cells, podocytes, and GECs. Abbreviations: PBMC: peripheral blood mononuclear cell; GMC: glomerular mesangial cell; GEC: glomerular endothelial cell.

**Supplementary Figure 2.** The expression profile of marker genes in four types of renal intrinsic cells. PDGFRB for GMCs, LRP2 for HK-2 cells, NPHS1 for podocytes, and CD31 for GECs. Abbreviations: GMC: glomerular mesangial cell; GEC: glomerular endothelial cell; PDGFRB: platelet derived growth factor receptor beta; LRP2: low-density lipoprotein receptor related protein 2.

**Supplementary Figure 3.** Complement components that were not detected in normal human renal tissues. These images were collected from the Human Protein Atlas (www.proteinatlas.org) after cropping the positive expression area from the original full images. Abbreviations: FCN1: ficolin 1; MASP1: MBL associated serine protease 1; C3AR1: complement C3a receptor 1.

**Supplementary Figure 4**. The expression of cell-specific markers in each cell type. The color intensity represents mean expression level and the dot size represents the percentage of expressing cells. Abbreviations: GMC: glomerular mesangial cell; PTEC: proximal tubular epithelial cell; GEC: glomerular endothelial cell; NK: natural killer.

**Supplementary Figure 5**. Dot plot showing both average expression and the percentage of positive cells among GECs, GMCs, podocytes, and PTECs. The color intensity represents mean expression level and the dot size represents the percentage of expressing cells. Abbreviations: GEC: glomerular endothelial cell; GMC: glomerular mesangial cell; PTEC: proximal tubular epithelial cell.

**Gel image of RT-PCR:**
